# The Immune-Enhancing Effects of a *Lactobacillus paracasei* L-30 Extract Through the NF-κB and MAPK Pathways in RAW264.7

**DOI:** 10.3390/cimb47020095

**Published:** 2025-02-03

**Authors:** Soyeon Kim, Inwook Kim, Sangkyu Park, Jeongmin Seo

**Affiliations:** Biomedical Research Institute, NeoRegen Biotech Co., Ltd., Suwon 16614, Republic of Korea; sotkfkd1685@naver.com (S.K.); infinite123@naver.com (I.K.)

**Keywords:** probiotics, *Lactobacillus paracasei*, macrophage activation, immunostimulatory, immune enhancer

## Abstract

Immune enhancement is an important factor that not only helps prevent infections but also affects overall health. This study aims to evaluate the immunostimulatory effects of a novel *Lactobacillus* strain, *Lactobacillus paracasei* L-30, and to elucidate its underlying mechanisms. The extract obtained from *Lactobacillus paracasei* L-30 significantly increased phagocytosis and the production of NO and ROS in RAW264.7 macrophages. The protein and mRNA expression levels of COX-2 and iNOS which are immune regulators were upregulated by the L-30 extract. The levels of cytokines and chemokines, such as G-CSF, IL-6, MIP-1α, MIP-1γ, RANTES, sTNF RI, and sTNF RII, were increased by the treatment with the L-30 extract. In addition, the L-30 extract degraded IκB-α and induced the phosphorylation of NF-κB. Furthermore, the MAPK signaling pathways ERK, JNK, and p38 were activated by the L-30 extract. The production of iNOS, COX-2, and NO was inhibited by MAPK pathway inhibitors. Therefore, our data suggest that the *Lactobacillus paracasei* L-30 extract has the potential to be developed as a healthy functional food that can enhance immune responses by activating macrophages.

## 1. Introduction

Immune enhancement is an important strategy that strengthens the body’s resistance to infections, diseases, and inflammation by promoting immune responses. In particular, immune responses can be enhanced through innate immunity, which is activated immediately by external pathogens [1]. Innate immune response constitutes the first line of defense, involving immune cells such as macrophages, NK cells, dendritic cells, and neutrophils [2,3]. Macrophages are widely distributed and activate immune cells by secreting a variety of chemicals that induce inflammatory responses and attack pathogens [4]. Activated macrophages eliminate invading pathogens through phagocytosis and the secretion of nitric oxide (NO) and reactive oxygen species (ROS) [5,6]. They also secrete various inflammatory mediators, such as interleukin-6 (IL-6), inducible nitric oxide synthase (iNOS), and cyclooxygenase-2 (COX-2), to activate immune responses [7]. 

Immunodeficiency is treated by strengthening the immune system using natural foods or medications [8]. However, the frequent use of medications can cause side effects such as headaches, fever, and hypertension [9,10]. Therefore, natural substances such as probiotics with fewer side effects are attracting attention. The WHO defines probiotics as “live microorganisms which, when administered in adequate amounts, confer a health benefit on the host” [11]. The probiotic market is growing significantly worldwide, with an expected compound annual growth rate of 14.1% from 2024 to 2030 [12]. Probiotics contribute to maintaining a healthy gut environment by promoting the growth of beneficial bacteria and inhibiting the growth of harmful bacteria [13]. In particular, *Lactobacillus* strains have been recognized for their safety by the WHO, and various studies have demonstrated that these strains influence gut health improvement, weight loss, and the enhancement of mental health [14,15,16]. Research on immunity is actively being conducted, and it is known that *Lactobacillus* plays an important role in improving immune function [17]. The immune-enhancing effects of these probiotics can be developed for applications in therapeutics and health functional foods. Therefore, the development of novel probiotic strains is important. 

*Lactobacillus paracasei* is associated with numerous health benefits. This strain has been the subject of various studies focusing on its antimicrobial action, anti-obesity properties, and mental health alleviation [18]. Additionally, *Lactobacillus paracasei* HII01 has been shown to extend lifespan and improve age-related factors in Caenorhabditis elegans [19]. Moreover, this strain has been reported to alleviate gastrointestinal disturbances associated with irritable bowel syndrome [20]. However, there is currently no research on the immune-enhancing effects of the *Lactobacillus paracasei* L-30 strain in macrophages and its underlying mechanisms. Therefore, we confirmed the immune-enhancing effects of the L-30 extract through the improvement in the phagocytic ability of macrophages. Furthermore, we observed the activation of macrophages and the associated mechanisms through an increase in immune-related substances and cytokines. In summary, we demonstrated that the extract of *Lactobacillus paracasei* L-30 enhances the immune response by inducing the activation of macrophages, which play a significant role in innate immunity.

## 2. Materials and Methods

### 2.1. Materials

Antibodies were purchased from the following suppliers: p-p38 (4511), p38 (9212), p-ERK (4370), ERK (9102), p-JNK (4668), JNK (9252), p-p65 (3033), and IκB-alpha (9242) were purchased from CST (Cell Signaling Technology, Boston, MA, USA). SP600125 (HY12041), PD98059 (HY12028), and SB203580 (HY10256) were obtained from MCE (MedChemExpress, Monmouth Junction, NJ, USA) for use in experiments. LPS (Sigma-Aldrich, St. Louis, MO, USA, L4391), iNOS (Novus, Littleton, CO, USA, NB300-605), COX-2 (Invitrogen, Waltham, MA, USA), and actin (GeneTex, Irvine, CA, USA, GTX629630) were purchased from their respective companies. 

### 2.2. Lactobacillus paracasei L-30 Culture and Extraction

The *Lactobacillus paracasei* L-30 strain (KCTC16035BP) was purchased from NeoRegen Biotech and cultured in MRS broth (Candalab, Ayer Rajah, Singapore 1215.00) at 35 °C for 18 h. The cultured L-30 strain was centrifuged at 10,000× *g* for harvesting. The L-30 pellet was washed three times with distilled water to remove the MRS broth. The washed L-30 pellet was resuspended in distilled water and sonicated for 30 min. The lysate was centrifuged at 10,000× *g* for 20 min to collect the supernatant. The supernatant was filtered using a 0.22 μm filter and lyophilized to obtain L-30 extract powder. For experimental use, the L-30 extract was dissolved in distilled water and stored at −20 °C. The L-30 extract was analyzed for protein concentration using a BCA protein assay kit (Thermo Fisher Scientific, Waltham, MA, USA, 23225), and the substances were treated based on the measured protein values. A 16S rRNA analysis of *Lactobacillus paracasei* L-30 was outsourced to Macrogen.

### 2.3. Cell Culture

Murine RAW264.7 macrophages were purchased from the American Type Culture Collection (ATCC, Manassas, VA, USA). The cells were cultured in Dulbecco’s Modified Eagle Medium (DMEM, Welgene, Gyeongsan, Korea, LM001-05) supplemented with 10% Fetal Bovine Serum (FBS, Millipore, MA, USA, TMS-013-BKR) and 1% penicillin/streptomycin (Waltham, MA, USA, 15140-122). We conducted our experiments using cells with passage numbers less than 20. The cells were cultured in an incubator at 37 °C in a humidified 5% CO_2_ atmosphere. The cells were sub-cultured at 70–80% confluence.

### 2.4. Cell Viability Assay

RAW264.7 was seeded in 96-well plates at a density of 5.0 × 10^3^ cells/well. After overnight incubation, the media were replaced with fresh media containing the L-30 extract, and the cells were treated for 24 h. Cell viability was assessed using an EZ-Cytox kit (DoGenBio, Seoul, South Korea, EZ-1000). After 24 h of treatment, 10 μL of the WST-8 solution was added to each well, and after one hour, the OD450 nm value was measured at 450 nm using a microplate reader.

### 2.5. ROS Production Assay

ROS production in RAW264.7 was evaluated using the ROS Detection Cell-Based Assay Kit (DCFDA, Cayman, Ann Arbor, MI, USA, 601520) according to the manufacturer’s instructions. Briefly, RAW264.7 was seeded in 96-well plates at a density of 1.0 × 10⁴ cells/well. The following day, the cells were treated with media containing the L-30 extract and LPS (1 μg/mL) for 24 h. Then, ROS staining buffer (10 μM) was added, and the cells were incubated at 37 °C for 90 min in the dark. Fluorescence was measured using a fluorescence plate reader (TECAN, Männedorf, Switzerland) with an excitation wavelength of 500 nm and an emission wavelength of 550 nm.

### 2.6. Phagocytosis Assay

RAW264.7 was seeded in 96-well plates at a density of 5.0 × 10⁴ cells/well. After overnight incubation, the cells were treated with the L-30 extract and LPS (1 μg/mL). After 24 h, the media were removed, and the cells were washed with PBS. Then, 100 μL of 0.1% neutral red solution (Sigma, N2889) in PBS was added, and the cells were incubated at 37 °C for 1 h. After incubation, the cells were washed with PBS three times and treated with a solution of acetic acid and ethanol (1:1). The cells were incubated at 4 °C for 2 h, and the absorbance was measured at 540 nm using a microplate reader.

### 2.7. NO Production Assay

RAW264.7 was grown in 96-well plates at a density of 1.0 × 10^5^ cells/well. The following day, the cells were treated with the L-30 extract and LPS (1 μg/mL) for 48 h. Nitric oxide (NO) levels in the culture supernatants were measured using the Griess reagent (Sigma, G4410). Equal volumes of supernatant and Griess reagent were mixed and incubated at room temperature (RT) for 10 min. The absorbance was measured at 540 nm using a microplate reader.

### 2.8. Western Blot Analysis

RAW264.7 was seeded into 6-well plates at a density of 5.0 × 10⁵ cells/well. After 18 h of incubation, the cells were treated with the L-30 extract and LPS (1 μg/mL) for 24 h. The cells were washed with PBS, and protein samples were prepared using a Luciferase Cell Culture Lysis 5X Reagent (Promega, Madison, WI, USA, E153A) supplemented with 1000X protease inhibitor and 1000X phosphatase inhibitor. Protein concentration was determined using a Pierce™ BCA Protein Assay Kit (Thermo, 23225). The extracted proteins were denatured by adding 5X loading buffer and boiling at 100 °C for 10 min. The proteins were separated via 12% SDS–polyacrylamide gel electrophoresis and transferred onto a polyvinylidene difluoride membrane. The membrane was blocked with 5% skim milk at RT for 2 h and incubated with primary antibodies overnight at 4 °C. We diluted 10X TBS with Tween-20 (Biosolution, Seoul, South Korea, BT007-1) to a 1X working solution. The composition of the 10X stock solution includes Tris at 247 mM, KCl at 27 mM, NaCl at 1.37 mM, and Tween-20 at 0.5%. After washing with 1X TBST, the membrane was incubated with secondary antibodies at RT for 2 h. The membrane was treated with an ECL solution, and the protein bands were visualized using a Bio-Image Analyzer (Viber Lourmat, Collégien, France). Inhibitors were pretreated at a concentration of 10 μM for 2 h, and the rest of the procedure was conducted as described above.

### 2.9. qRT-PCR Analysis

RAW264.7 was seeded at a density of 5.0 × 10⁵ cells per well in 6-well plates. The following day, the cells were treated with the L-30 extract and LPS (1 μg/mL) for 24 h. Total RNA was extracted using a MiniBEST Universal RNA Extraction Kit (TaKaRa, Shiga, Japan, 9767). RNA concentration was measured using a Nanodrop. cDNA was synthesized from RNA using a TOPscript RT DryMIX kit (dT18; Enzynomics, Daejeon, South Korea, RT200). The cDNA was amplified and analyzed using TB Green Premix Ex Taq II (TaKaRa, RR820A) and a 7500 Real-Time PCR System (Applied Biosystems, Waltham, MA, USA). The primers used are summarized in Table 1. GAPDH was used as a housekeeping gene to normalize differences between samples.

### 2.10. Cytokine Array

RAW264.7 was grown in 60 mm dishes at a density of 5.0 × 10⁵ cells/dish. The following day, the cells were treated with the L-30 extract for 48 h. Culture supernatants were collected and stored at −80 °C until use. Cytokine levels were assessed using a Mouse Inflammation Antibody Array Membrane (Abcam, Cambridge, UK, AB133999). Images were captured using a Bio-Image Analyzer (Viber Lourmat, Collégien, France), and cytokine quantification was performed using ImageJ (Version 1.54K).

### 2.11. Statistics

All measurements were conducted in triplicate, and the results are presented as the mean ± standard deviation. Differences between the results were assessed for significance using Student’s *t*-test. * *p* < 0.05 and ** *p* < 0.01 indicate a statistically significant difference compared to the control.

## 3. Results

### 3.1. Identification of Lactobacillus paracasei L-30

**Figure 1:**
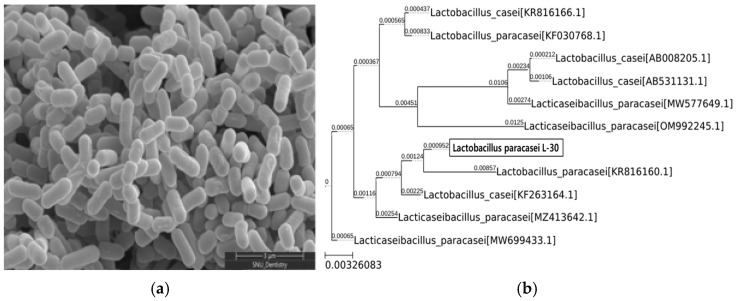
SEM image and phylogenetic tree of *Lactobacillus paracasei* L-30. (**a**) Scanning electron microscopy image of *Lactobacillus paracasei* L-30; scale bar represents 3 μm (X30,000). (**b**) Phylogenetic tree based on 16S rRNA gene sequencing of *Lactobacillus paracasei* L-30.

The morphology of the *Lactobacillus paracasei* L-30 strain was observed using scanning electron microscopy (Figure 1a). To confirm the identification of the *Lactobacillus paracasei* L-30 strain, a 16S rRNA gene analysis was conducted. The results showed a 99.93% sequence identity with the *Lactobacillus paracasei* species (Figure 1b).

### 3.2. Effects of L-30 Extract on Cell Viability and ROS Production in RAW264.7

**Figure 2:**
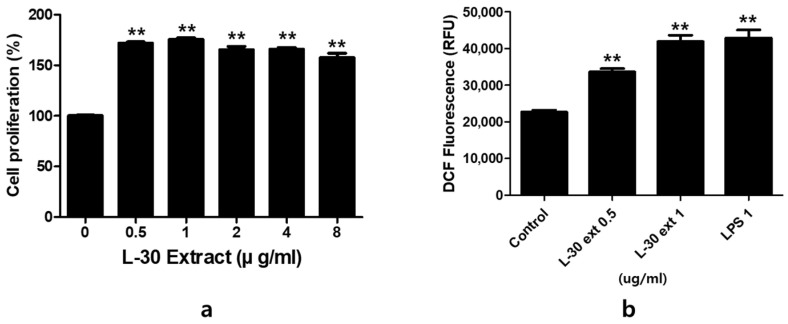
The effects of L-30 extract on cytotoxicity and ROS production in RAW264.7 macrophages. (**a**) The cells were treated with the L-30 extract (0, 0.1, 1, 2, 4, 8 µg/mL) for 24 h, and viability was then quantified using a CCK-8 assay. (**b**) An ROS Detection kit was used to establish the ROS production of the L-30 extract (L-30 Ext). The results show the mean ± standard deviation of three experiments. ** *p* < 0.01 indicates a statistically significant difference compared to the control.

To evaluate the cytotoxicity of the L-30 extract, RAW264.7 was cultured for 24 h with concentrations of 0, 0.5, 1, 2, 4, and 8 µg/mL. The results indicated that at concentrations of the L-30 extract greater than 0.5 µg/mL, cell viability increased by over 70% compared to the control group (Figure 2a). The effects of the L-30 extract on ROS production in RAW264.7 were examined. Following a 24 h treatment with the L-30 extract, the level of ROS generated in macrophages was significantly increased in a dose-dependent manner compared to the control group (Figure 2b). We used lipopolysaccharide (LPS) as a positive control. These results suggest that the L-30 extract is non-cytotoxic and the activation of macrophages increases the generation of ROS.

### 3.3. Effects of L-30 Extract on Phagocytosis and NO Production in RAW264.7

**Figure 3:**
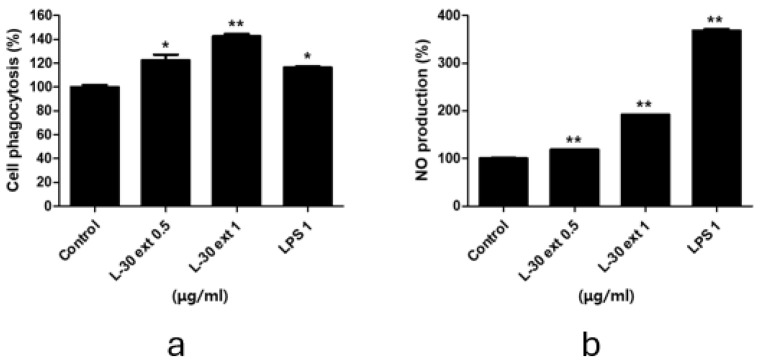
L-30 extract increased phagocytotic and nitric oxide production in RAW264.7 macrophages. (**a**) Phagocytosis activity of L-30 extract was determined by uptake of neutral red. (**b**) NO production of L-30 extract was examined using Griess reagent. Results show mean ± standard deviation of three experiments. * *p* < 0.05 and ** *p* < 0.01 indicate statistically significant difference compared to control.

Phagocytosis is a critical and essential process for the removal of pathogens and cell debris. To evaluate phagocytic activity, the amount of neutral red uptake by macrophages treated with the L-30 extract for 24 h was assessed. LPS was used as a positive control in phagocytosis and NO experiments. As shown in Figure 3a, treatment with the L-30 extract resulted in a significant increase in phagocytic activity compared to the control group. Moreover, phagocytic capacity was further enhanced with increasing concentrations of the L-30 extract. In addition, the L-30 extract exhibited a greater phagocytic capacity compared to the positive control. These results showed that macrophages activated by the L-30 extract exhibit elevated phagocytic ability, effectively defending against pathogens. The pathogens engulfed by macrophages are damaged structurally by NO and destroyed. NO acts as an immune signaling molecule that activates immune cells. Therefore, we examined NO production induced by the L-30 extract using a Griess assay. The results demonstrated that NO production increased in a dose-dependent manner with L-30 extract treatment (Figure 3b). This finding confirms that the elevated NO levels contribute to immune enhancement by activating macrophages.

### 3.4. Effects of L-30 Extract on Protein and mRNA Expression of COX-2 and iNOS in RAW264.7

**Figure 4:**
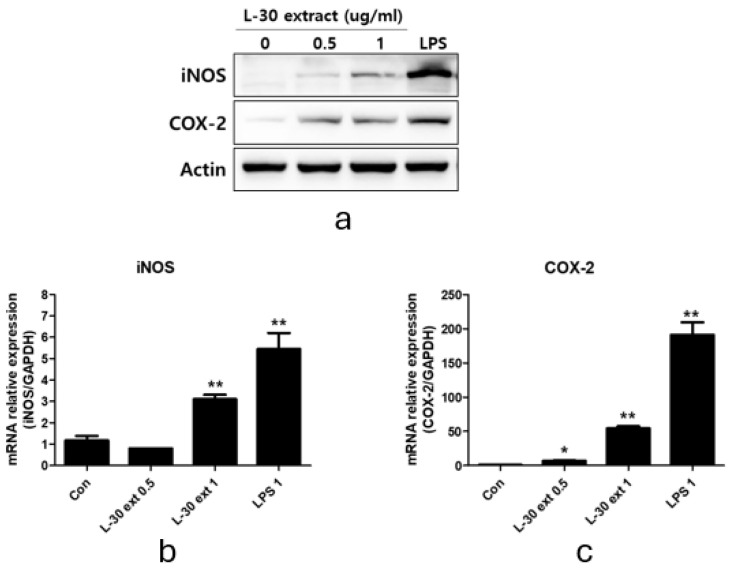
L-30 extract promoted iNOS and COX-2 expression in RAW264.7 macrophages. (**a**) Quantification of protein levels by Western blot analysis. Quantification of (**b**) iNOS and (**c**) COX-2 mRNA levels by qRT-PCR. Results show mean ± standard deviation of three experiments. * *p* < 0.05 and ** *p* < 0.01 indicate statistically significant difference compared to control.

To explore macrophage activation, we evaluated the protein and mRNA expression levels of COX-2 and iNOS, which play an important role in immune enhancement. iNOS is the primary enzyme responsible for NO production, while COX-2 is involved in PGE2 synthesis. RAW264.7 was treated with the L-30 extract at concentrations of 0.5 and 1 µg/mL, and 1 µg/mL LPS was used as a positive control for 24 h. The Western blot results showed that the protein expression levels of iNOS and COX-2 increased relative to the control group (Figure 4a). The 0.5 µg/mL concentration of the L-30 extract showed no change in iNOS mRNA levels, but using the L-30 extract of 1 µg/mL showed a significantly increase in these levels (Figure 4b). In addition, COX-2 mRNA levels were elevated in a dose-dependent manner at concentrations of 0.5 and 1 µg/mL (Figure 4c). These findings indicate that the L-30 extract enhances the production of both COX-2 and iNOS at the protein level, as well as increasing their mRNA expression.

### 3.5. The L-30 Extract Induces the Secretion of Cytokines and Chemokines in RAW264.7

**Figure 5:**
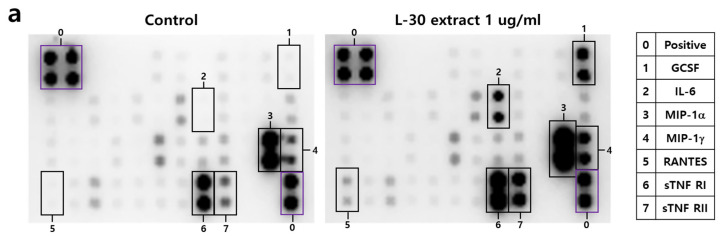

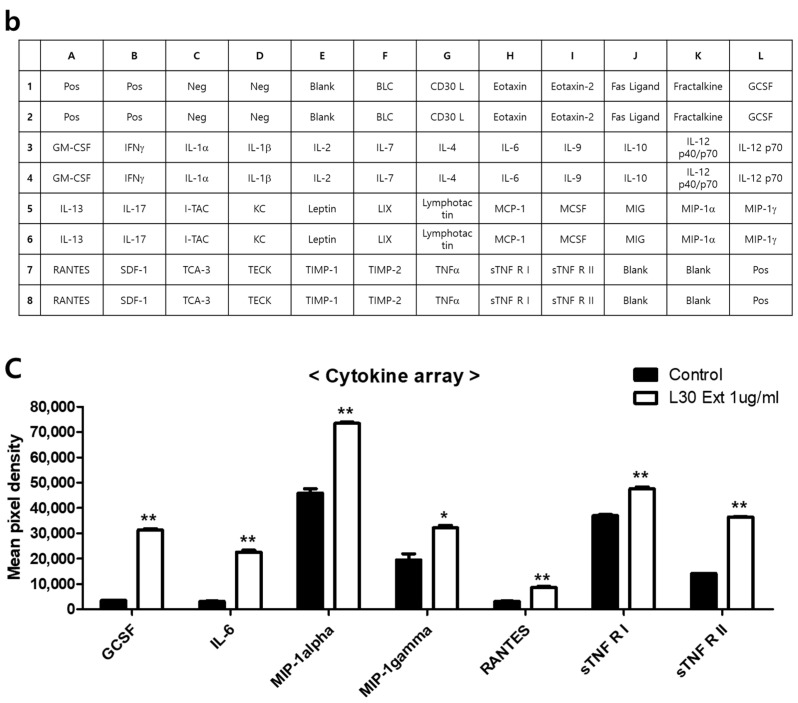
The effects of the L-30 extract on a cytokine array in RAW264.7 macrophages. (**a**) The two membranes were visualized using a CCD camera. (**b**) The positions of the cytokine spots on the membrane are represented. (**c**) The membranes of cytokines were quantified using Image J. The results show the mean ± standard deviation of two experiments. * *p* < 0.05 and ** *p* < 0.01 indicate a statistically significant difference compared to the control.

To investigate the cytokines and chemokines secreted by macrophages in response to the L-30 extract, a mouse inflammation cytokine array kit was used to evaluate the trends in 40 cytokines. Among the 40 antibodies, 7 cytokines were significantly altered by the L-30 extract (Figure 5a,b). These cytokines include G-CSF, IL-6, MIP-1α, MIP-1γ, RANTES, sTNF RⅠ, and sTNF RⅡ (Figure 5c). Granulocyte colony-stimulating factor (G-CSF) and IL-6 are substances that activate immune cells by regulating cytokines and inflammatory responses, and the L-30 extract increased both. Macrophage Inflammatory Protein-1 alpha (MIP-1α), Macrophage Inflammatory Protein-1 gamma (MIP-1γ), and RANTES (CCL5) are chemokines that function to attract immune cells to sites of inflammation or infection, and the levels of these were also elevated by the L-30 extract compared to the control. The levels of Soluble Tumor Necrosis Factor Receptor I (sTNF RⅠ) and sTNF RⅡ, which are involved in regulating the TNF pathway, were increased by the L-30 extract as well. Therefore, the L-30 extract showed its potential as an immunostimulatory agent by inducing inflammatory molecules such as G-CSF, IL-6, MIP-1α, MIP-1γ, RANTES, sTNF RⅠ, and sTNF RⅡ.

### 3.6. L-30 Extract Activates MAPK and NF-κB Signaling Pathways in RAW264.7

**Figure 6:**
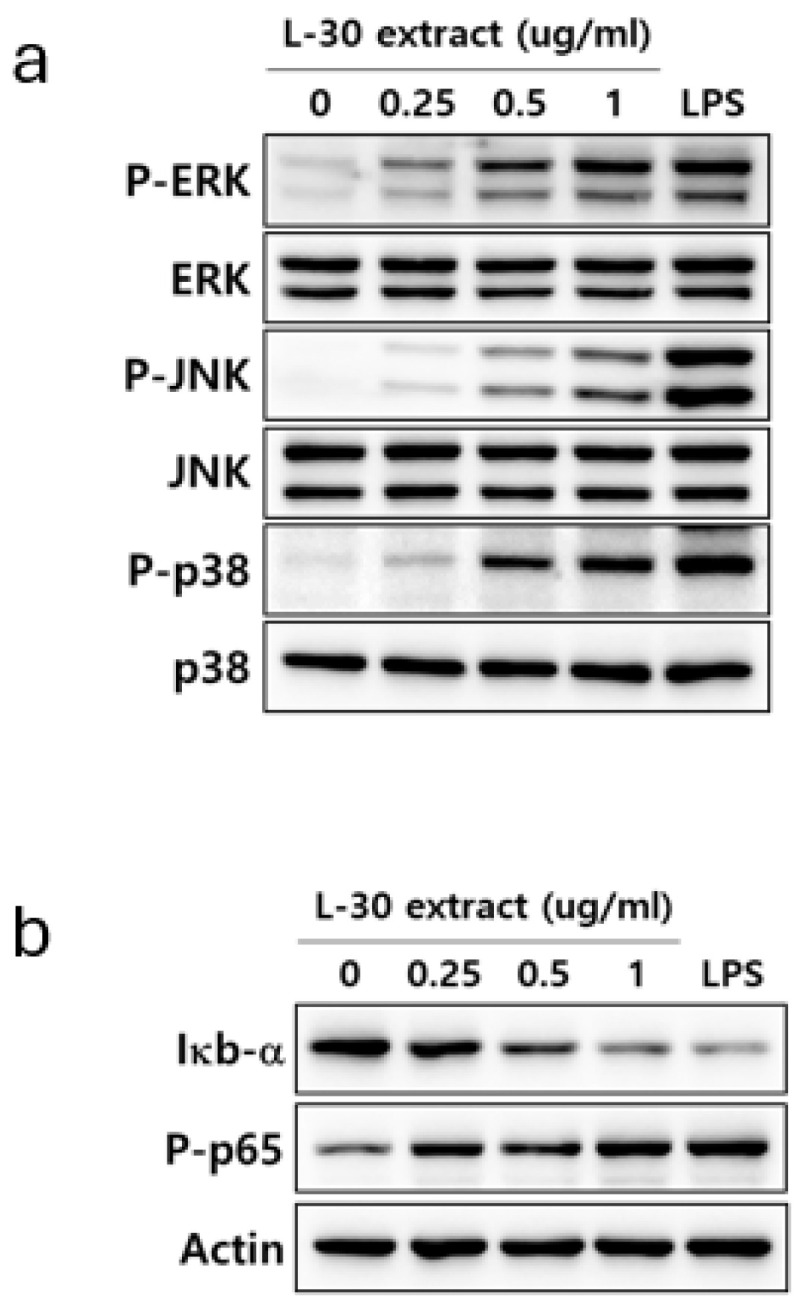
Effects of L-30 extract on MAPK and NF-κB pathways in RAW264.7 macrophages. (**a**) MAPK pathway and (**b**) NF-κB pathway were assessed by Western blot assay. LPS was used as positive control and was treated at concentration of 1 µg/mL for 30 min. RAW264.7 was treated with L-30 extract at concentrations of 0.25, 0.5, and 1 µg/mL for 30 min.

To elucidate the mechanism by which the L-30 extract activates the immune response, the activities of MAPK and NF-κB signaling were confirmed. LPS was used as a positive control for the activation of the MAPK and NF-κB pathways. RAW264.7 was treated with the L-30 extract at concentrations of 0.25, 0.5, and 1 µg/mL, along with 1 µg/mL of LPS for 30 min. Treatment with the L-30 extract resulted in a dose-dependent increase in the phosphorylation of ERK, JNK, and p38 compared to the control group (Figure 6a). To investigate the effects of the L-30 extract on the NF-κB signaling pathway, the degradation of IκB-α and the phosphorylation of p65 were assessed. The results showed that IκB-α was degraded by the L-30 extract, with a dose-dependent reduction. Furthermore, treatment with the L-30 extract led to an increase in p65 phosphorylation, demonstrating the activation of the NF-κB pathway (Figure 6b). These findings suggest that the activation of macrophages by the L-30 extract is mediated through the MAPK and NF-κB signaling pathways.

### 3.7. Immune-Enhancing Effects Induced by L-30 Extract Were Blocked by MAPK Pathway Inhibitor

**Figure 7:**
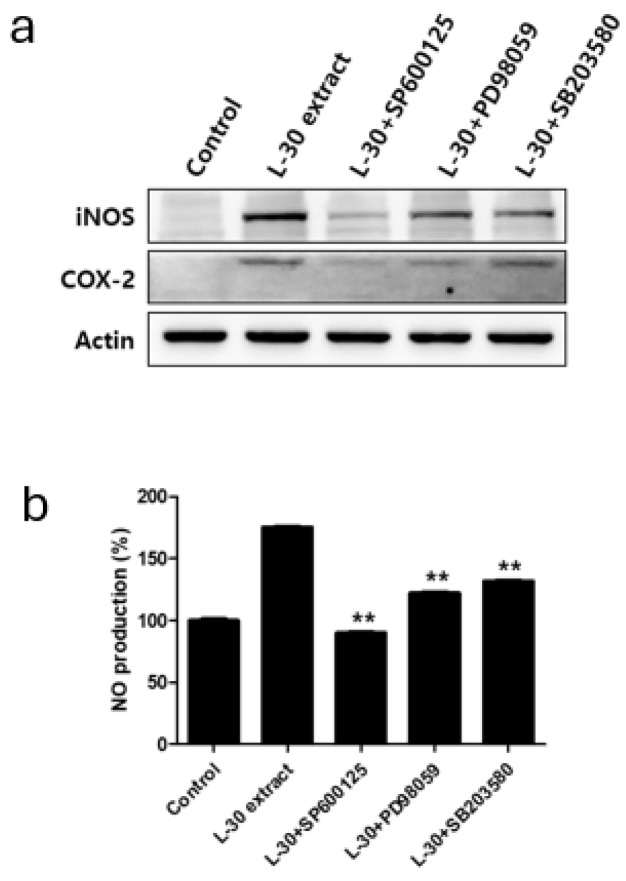
Effects of MAPK pathway inhibitor on immunostimulatory markers in RAW264.7 macrophages. (**a**) SP600125 (JNK inhibitor), PD98059 (ERK inhibitor), and SB203580 (p38 inhibitor) were pretreated for 2 h, and then L-30 extract (1 µg/mL) was treated for 24 h. Expression of iNOS and COX-2 was evaluated using Western blot analysis. (**b**) NO production of MAPK pathway inhibitor was examined using Griess reagent. Results show mean ± standard deviation of three experiments. ** *p* < 0.01 indicates statistically significant difference compared to L-30 extract.

Specific pathway inhibitors, including the JNK inhibitor SP600125 (10 µM), the ERK inhibitor PD98059 (10 µM), and the p38 inhibitor SB203580 (10 µM), were used to determine whether the immune-enhancing effects induced by the L-30 extract were regulated by the MAPK pathway. The inhibitors were pretreated for 2 h, followed by treatment with the L-30 extract for 24 h. Western blot analysis revealed that the MAPK pathway inhibitors effectively inhibited the production of iNOS and COX-2 induced by the L-30 extract (Figure 7a). In addition, it was observed that the increase in NO levels induced by the L-30 extract was reduced in RAW264.7 treated with the three inhibitors (Figure 7b). These results show that the immune-enhancing effects of the L-30 extract are regulated by the MAPK signaling pathway.

## 4. Discussion

Immunity is an important physiological response that protects the body from the outside, and lactic acid bacteria (LAB) have recently been investigated as a substance for maintaining and increasing immunity [21]. This is because LAB are microorganisms that are naturally present in the human body and have a low risk of side effects, and studies have shown that they can improve long-term immune function by balancing the immune system [8]. While *Lactobacillus* is related to immunity, *Lactobacillus paracasei* is attracting attention for its excellent intestinal colonization ability and high survival rate in gastric acid and bile acid [22,23]. *Lactobacillus paracasei* KW3110 has been demonstrated to inhibit allergic responses by modulating dendritic cells and Th2 cells [24]. In addition, *Lactobacillus paracasei* ST11 contributes to immunomodulatory responses through the production of IL-10 by stimulating regulatory T cells [25]. As described above, *Lactobacillus paracasei* has been reported to have various immune-related functions; however, it is essential to develop novel strains and conduct further research. This is necessary because different strains of the same species can play varying roles in the immune response. We isolated *Lactobacillus paracasei* L-30 from the saliva of breastfed newborns and confirmed its immune-enhancing effects. Therefore, we developed this study to analyze the immune-stimulating properties and mechanisms of the newly discovered L-30 in macrophages to show that L-30 has potential as a therapeutic and health functional food in the future.

We focused on whether the L-30 extract enhances phagocytic functions. Phagocytosis plays an important role in the direct elimination of pathogens and the activation of macrophages [26]. Our results indicate that the L-30 extract enhances phagocytic activity, suggesting that this leads to the induction of the innate immune response through the activation of macrophages. In addition, the expression of iNOS and COX-2 was evaluated to confirm the macrophage activation effects of the L-30 extract. iNOS and COX-2 produced by activated macrophages are important enzymes that play roles as immunostimulatory mediators [27]. iNOS promotes the synthesis of NO, and the generated NO interacts with ROS to directly destroy pathogens [28,29]. Pro-inflammatory cytokines such as IL-6 also play a crucial role in the immune response by not only activating macrophages in the innate immune response but also supporting adaptive immune responses [30,31]. As shown in Figure 4, the L-30 extract upregulated important proteins in the immune response, such as iNOS and COX-2. Furthermore, it was confirmed that the L-30 extract contributes to immune enhancement by promoting the production of the pro-inflammatory cytokine IL-6 using the cytokine array.

In this study, the L-30 extract increased the levels of MIP-1α, MIP-1γ, RANTES, GCSF, sTNF RⅠ, and sTNF RⅡ through a cytokine array (Figure 5). Chemokines such as MIP-1α, MIP-1γ, and RANTES are essential proteins that recruit immune cells to the site of inflammation, which plays a role in enhancing immune responses [32]. In addition, G-CSF activates neutrophils, which are important in the innate immune response, detecting various pathogens, and remove pathogens through phagocytosis [33]. The production of chemokines and G-CSF activates not only macrophages but also other immune cells. The L-30 extract also increased the production of sTNF RⅠ and sTNF RⅡ, which regulate the immune response. sTNF RⅠ and sTNF RⅡ are present in fluids such as plasma and modulate their activity by binding with TNF-α [34]. In particular, sTNF RⅡ is known to play a role in regulating excessive immune responses [35]. It was reported that ovotransferrin induces immune enhancement under normal conditions but provides immune regulation in cases of overactive immunity [36]. Similarly, the L-30 extract showed the potential to enhance immunity under normal conditions while inducing sTNFR to regulate immunity in overactive immune states. 

Our results showed that the L-30 extract increased the levels of cytokines that induce immune responses through the NF-κB signaling pathway in macrophages (Figure 6b). Normally, NF-κB binds to the IκB-a inhibitory protein in the cytoplasm to form an IκB kinase complex [37]. When the NF-κB pathway is activated by a stimulus, IκB-a is released via ubiquitination and proteasome degradation. Phosphorylated NF-κB is then translocated to the nucleus, where it activates the expression of immune-related cytokine genes to induce an immune response [38]. As shown in Figure 6b, the L-30 extract induced the phosphorylation of NF-κB through the degradation of IκB-α, which activated macrophages. Several studies have shown that the activation of NF-κB is promoted by the MAPK pathway, and MAPK is closely involved in immune function [39]. It has been reported that WE-HML activates the JNK pathway and induces the expression of iNOS, COX-2, and IL-6 to enhance immune functionality [40]. In addition, SCOP was found to activate macrophages through the production of various cytokines via the phosphorylation of JNK, ERK, and p38 [4]. Other studies have shown that NF-κB is activated by the MAPK pathway, resulting in an enhanced expression of immune-related cytokines [41]. As shown in Figure 6, our results also showed that the L-30 extract promoted the activation of JNK, ERK, and p38. In addition, we verified that the immune enhancement induced by the L-30 extract was completely blocked when the MAPK inhibitor was used. Therefore, we concluded that the L-30 extract activates macrophages through the MAPK-mediated NF-κB signaling pathway.

When macrophages are activated, they induce an immune response via pattern recognition receptors (PPRs) such as Toll-like receptors (TLRs), advanced glycation end products (RAGEs), and NOD-like receptors (NLRs) [42]. In particular, activated TLR4 has been reported to enhance the body’s immunity by inducing the phosphorylation of MAPKs, which translocates NF-κB to the nucleus to produce immune-related cytokines [43]. In another study, *Lactobacillus* strains were found to activate the NF-κB pathway via TLR4 to activate the immune response [44]. In addition, exopolysaccharides (EPSs) isolated from *Lactobacillus paracasei* stimulated the immune response by activating TLR4 and inducing the phosphorylation of MAPK and NF-κB [45]. Based on the above results, we hypothesized that the L-30 extract acts on TLR4 to activate the downstream signaling MAPK and NF-κB pathways. 

There are some limitations to this study. Further research is required to identify the specific component responsible for immune enhancement, as the L-30 extract is a mixture of various components. It is necessary to determine whether the observed effects are due to distinct immunomodulatory components or simply a response to pathogen-associated molecular patterns (PAMPs) recognized by pattern recognition receptors (PRRs). Additionally, identifying the primary receptor through which the L-30 extract induces immune enhancement would help better understand its cellular uptake process. Furthermore, the immune-enhancing effects of the L-30 extract were demonstrated in vitro, but its ability to maintain immune balance without inducing excessive inflammation remains uncertain. These issues should be addressed through further in vivo studies. Once these issues are further addressed, a more thorough confirmation of the immune-enhancing effects of the L-30 extract will be possible.

Despite these limitations, our findings highlight the importance of the L-30 extract, a specific probiotic, in inducing immune enhancement. This research was conducted using macrophages, which play a crucial role in the early stages of the immune response. It was confirmed that the L-30 extract induces the activation of macrophages, demonstrating its potential as an immune-enhancing functional ingredient. These findings provide an important foundation for uncovering the mechanism of action of the L-30 extract in macrophages and will serve as a basis for further advancing future research. 

## 5. Conclusions

**Figure 8:**
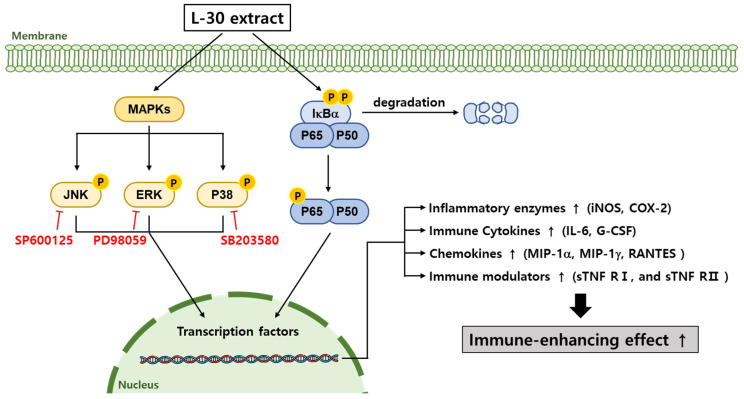
Schematic diagram of MAPK and NF-κB signaling pathways in terms of macrophages induced via L-30 extract. When L-30 extract is treated to macrophages, IκB-α is degraded, and P65 is phosphorylated. In addition, MAPK pathways are phosphorylated to promote immune-related gene expression. As a result, expression of COX-2 and iNOS mRNA is induced to produce NO, thereby activating macrophages. In addition, L-30 extract induces immune responses by upregulating immune-related cytokines and chemokines.

The L-30 extract was confirmed to have immune-enhancing effects in RAW264.7 macrophages (Figure 8). The L-30 extract is non-toxic and removes pathogens by activating phagocytosis in macrophages. It not only enhanced the immune response by increasing the expression of immune-related enzymes such as iNOS and COX-2 but also promoted the production of cytokines and chemokines. The immunostimulatory effects of the L-30 extract were shown to be induced by the activation of the MAPK and NF-κB signaling pathways. These effects were achieved by inducing the translocation of NF-κB through the degradation of IκB-α. Based on these results, it is suggested that the L-30 extract may serve as a functional raw material that helps enhance immunity in RAW264.7 macrophages. We plan to fractionate the L-30 extract to isolate its active component and then determine which receptor is involved in completing the signaling pathway of L-30-induced immune enhancement. Additionally, the L-30 extract will be evaluated in vivo to assess its potential as an immune enhancer and to determine whether it maintains adequate immune levels.

## Figures and Tables

**Table 1:** Primer sequences used for qPCR.

Gene	Forward primer (5’→3’)	Reverse primer (5’→3’)
COX-2	CAGACAACATAAACTGCGCCTT	GATACACCTCTCCACCAATGACC
iNOS	ATGTCCGAAGCAAACATCAC	TAATGTCCAGGAAGTAGGTG
GAPDH	ATGACTCCACTCACGGCAAA	GGGTCTCGCTCCTGGAAGAT

## Data Availability

Data are available upon request from the corresponding author.
